# Supplementary material for: Emergence of the London Millennium Bridge instability without synchronisation
Source: Nat Commun. 2021 Dec 10;12:7223. doi: 10.1038/s41467-021-27568-y (PMC8664840; doi:10.1038/s41467-021-27568-y)
Supplement: Supplementary file 1 — Supplementary Information [file 41467_2021_27568_MOESM1_ESM.pdf]

Supplementary information for

Emergence of the London Millennium Bridge instability without synchronisation

Igor Belykh, Mateusz Bocian, Alan R. Champneys, Kevin Daley, Russell Jeter, John H.G. Macdonald, and Allan McRobie

Supplementary Note 1: details of the asymptotic calculation of $\sigma_{1,2,3}$

We start from (22) (see the main paper). For ease of notation, we shall let $\dot{x} = u$, $\dot{y} = v$, $\dot{z} = w$ and drop the superscript (i) in what follows, providing the meaning is clear. Also, let us define the vector $\xi = (x,u,y,v,z,w)$ and let $\xi_0 = (0,0,y_0,v_0,\chi t + z_0, c + w_0)$ be the unperturbed limit-cycle motion. Now we can formally expand the functions H and G as power series

$$H = h_0 + \sum_{k=1}^{6} h_{\xi_k}(\xi_k - \xi_{k0}) + \mathscr{O}(|\xi - \xi_0|^2), \qquad G = g_0 + \sum_{k=1}^{6} g_{\xi_k}(\xi_k - \xi_{k0}) + \mathscr{O}(|\xi - \xi_0|^2). \tag{25}$$

Note that the coefficients h_{ξ_k}, and g_{ξ_k} represent partial derivatives of H and G with respect to their subscripted arguments, evaluated along the unperturbed solution $y = y_0(t)$, $z = \chi t + z_0(t)$. Hence each of these coefficients is a T-periodic function of time.

1.1 First-order solution

Substitution of the zeroth-order solution into (20) yields, to leading order,

$$\ddot{x}_1 + \Omega^2 x_1 = 0, \tag{26}$$

where we have used the assumption (21) that the pedestrians are uncorrelated to assume

$$\sum_{i=1}^{N} h_0^{(i)}(t) \ll \mathscr{O}(\varepsilon^{-1}).$$

The solution to (26) is the free vibration of the bridge, which can be written in the form

$$x_1(t) = X(\tau)\cos(\Omega t + \phi(\tau)), \tag{27}$$

where the amplitude X and phase ϕ are allowed to be functions of a slow time variable $\tau = \varepsilon t$.

Substitution of x_1 into (18) and (19) using (25) yields, to leading-order in ε,

$$\begin{pmatrix} \ddot{y}_1 \\ \ddot{z}_1 \end{pmatrix} + \begin{pmatrix} h_y & h_z \\ g_y & g_z \end{pmatrix} \begin{pmatrix} y_1 \\ z_1 \end{pmatrix} + \begin{pmatrix} h_v & h_w \\ g_v & g_w \end{pmatrix} \begin{pmatrix} \dot{y}_1 \\ \dot{z}_1 \end{pmatrix} = \begin{pmatrix} \Omega^2 - h_x & \Omega h_u \\ 0 & 0 \end{pmatrix} \begin{pmatrix} X(\tau)\cos[\Omega t + \phi(\tau)], \\ X(\tau)\sin[\Omega t + \phi(\tau)]. \end{pmatrix}. \tag{28}$$

This is a linear system with periodic coefficients and periodic forcing. It can be solved as the sum of free and forced vibration terms. Under the assumption that $\omega_i \neq \Omega$, and that the limit cycle in the absence of bridge motion is asymptotically stable, the free vibration part must decay to zero for large times. The only non-decaying part comes from the forced vibration. We find approximate expressions for this term by averaging the periodic functions h_ξ and g_ξ. Let an overbar represent the average of a quantity over each period T. That is

$$\bar{h}^{(i)}_{\xi_k} := \frac{1}{T_i}\int_0^{T_i} \left.\frac{\partial H^{(i)}}{\partial \xi_k}\right|_{x=0, y=y_0^{(i)}, z=\chi t + z_0^{(i)}} dt, \quad \bar{g}^{(i)}_{x_k} := \frac{1}{T_i}\int_0^{T_i} \left.\frac{\partial G^{(i)}}{\partial \xi_k}\right|_{x=0, y=y_0^{(i)}, z=\chi t + z_0^{(i)}} dt.$$

Then the solution for the forced vibration problem can be written in the form

$$y_1(t) = X(\tau)(y_c\cos[\Omega t + \phi(\tau)] + y_s\sin[\Omega t + \phi(\tau)] + y_r(t)), \tag{29}$$

$$z_1(t) = X(\tau)(z_c\cos[\Omega t + \phi(\tau)] + z_s\sin[\Omega t + \phi(\tau)] + z_r(t)) \tag{30}$$

where $y_{c,s}$, $z_{c,s}$ are constant amplitudes of cosines and sines of period T, and y_r and z_r are remainder terms that contain all other harmonics. Expressions for $y_{c,s}$, $z_{c,s}$ can be written in closed form as

$$y_c = \frac{D\Omega^2 - D\bar{h}_x - B\Omega\bar{h}_u}{AD - BC}, \qquad y_s = \frac{A\Omega\bar{h}_u - C\Omega^2 + C\bar{h}_x}{AD - BC},$$

$$z_c = -Z_2 y_c + Z_1 y_s, \qquad z_s = -Z_2 y_c + Z_1 y_s$$

where

$$A = \overline{h}_y - \Omega^2 - Z_1\overline{h}_z - Z_2\Omega\overline{h}_w, \qquad B = \Omega\overline{h}_v - Z_1\Omega\overline{h}_w + \overline{h}_z Z_2,$$

$$C = -\Omega\overline{h}_v + \overline{h}_w\Omega Z_1 - \overline{h}_z Z_2, \qquad D = \overline{h}_y - \Omega^2 - \overline{h}_w\Omega Z_2 - \overline{h}_z Z_1,$$

$$Z_1 = \frac{\Omega^2(\overline{g}_v\overline{g}_w - \overline{g}_y) + \overline{g}_y\overline{g}_z}{\Omega^4 + \omega^2\overline{g}_w^2 - 2\Omega^2\overline{g}_z + \overline{g}_z^2} \qquad Z_2 = \frac{\Omega^2\overline{g}_v - \Omega(\overline{g}_y\overline{g}_w - \overline{g}_v\overline{g}_z)}{\Omega^4 + \omega^2\overline{g}_w^2 - 2\Omega^2\overline{g}_z + \overline{g}_z^2}.$$

1.2 Second-order solution

Substitution of the $\mathcal{O}(1)$ solution into bridge equation (20) at second order yields

$$\ddot{x}_2 + \Omega^2 x_2 = \sum_{i=1}^{N} h_0^{(i)} + \varepsilon \sum_{i=1}^{N} h_1^{(i)} - [\dot{x}_1' + 2\zeta\Omega\dot{x}_1], \tag{31}$$

where $'$ means differentiation with respect to the slow time τ and

$$h_1^{(i)} = h_x^{(i)} x_1 + h_u^{(i)}\dot{x}_1 + h_y^{(i)} y_1^{(i)} + h_v^{(i)}\dot{y}_1^{(i)} + h_z^{(i)} z_1^{(i)} + h_w^{(i)}\dot{z}_1^{(i)}.$$

We can now substitute the form of x_1 from (27) and of $y_1^{(i)}$ and $z_1^{(i)}$ from (30) for each pedestrian i, and seek the general solution to the forced vibration problem.

In order to find a consistent asymptotic solution, under the formalism of the method of multiple scales[1], we must avoid secular terms on the right-hand side of (31). That is, the components of $\cos(\Omega t + \phi)$ and $\sin(\Omega t + \phi)$ in the forcing term must vanish. Let us consider the three terms on the right-hand side (31) in turn.

Consider first the term $\sum_{i=1}^{N} h_0^{(i)}$. Here the assumption (21) about frequency separation means that there can be no contribution to the secular terms from this sum. Next consider the term $\varepsilon\sum_{i=1}^{N} h_1^{(i)}$. At first this seems to be at lower order and so is unlikely to contribute. But, recalling the scaling (17) that $N = \mathcal{O}(1/\varepsilon)$ it may be that there is a cumulative contribution from each term in the sum that can contribute at the required order. By performing a spectral decomposition of the term $h_1^{(i)}$ we find the components of

$\cos(\Omega t+\phi)$ and $\sin(\Omega t+\phi)$ to be

$$\cos(\Omega t+\phi): \qquad X\left[\overline{h}_x^{(i)} + (\overline{h}_y^{(i)} y_c^{(i)} - \Omega \overline{h}_v^{(i)} y_s^{(i)}) + (\overline{h}_z^{(i)} z_c^{(i)} - \Omega \overline{h}_w^{(i)} z_s^{(i)})\right] = X\left[\overline{h}_x^{(i)} + \overline{\kappa}_y^{(i)} + \overline{\kappa}_z^{(i)}\right],$$
$$\sin(\Omega t+\phi): \; X\left[-\Omega\overline{h}_u^{(i)} + (\overline{h}_y^{(i)} y_s^{(i)} + \Omega \overline{h}_v^{(i)} y_c^{(i)}) + (\overline{h}_z^{(i)} z_c^{(i)} + \Omega \overline{h}_w^{(i)} z_c^{(i)})\right] = X\left[-\Omega\overline{h}_u^{(i)} + \overline{\sigma}_y^{(i)} + \overline{\sigma}_z^{(i)}\right].$$

Note that each of these coefficients is a constant for each pedestrian, because we already averaged out the period-T_i components in the definition (30). Therefore we can sum each of these N terms individually so that

$$\varepsilon\sum_{i=1}^{N}\left[\overline{h}_x^{(i)} + \overline{\kappa}_y^{(i)} + \overline{\kappa}_z^{(i)}\right] = \varepsilon N\left[\hat{h}_x + \hat{\kappa}_y + \hat{\kappa}_z\right], \;\; \varepsilon\sum_{i=1}^{N}\left[\overline{h}_u^{(i)} + \overline{\sigma}_y^{(i)} + \overline{\sigma}_z^{(i)}\right] = \varepsilon N\left[-\Omega\hat{h}_u + \hat{\sigma}_y + \hat{\sigma}_z\right],$$

where $\hat{\cdot}$ means averaging over all pedestrians

$$\hat{h}_x = \frac{1}{N}\sum_{i=1}^{N}\overline{h}_x^{(i)}, \quad \hat{h}_u = \frac{1}{N}\sum_{i=1}^{N}\overline{h}_u^{(i)}, \quad \text{etc.}$$

Finally, the third term on the right-hand side of (31) is

$$\dot{x}_1' + 2\zeta\Omega\dot{x}_1 = -\Omega X'(\tau)\sin(\Omega t+\phi(\tau)) - \phi' X\Omega\cos(\Omega t+\phi(\tau)) - 2\zeta X\Omega^2\sin(\Omega t+\phi(\tau)).$$

Recalling that $N\varepsilon = \nu$, the vanishing of the secular terms on the right-hand side of (31) thus implies

$$\begin{aligned} &\text{component of } \cos(\Omega t+\phi): \qquad 0 = \Omega\phi' X + \nu X\left[\hat{h}_x + \hat{\kappa}_y + \hat{\kappa}_z\right],\\ &\text{component of } \sin(\Omega t+\phi): \qquad 0 = \Omega X'(\tau) + 2\zeta X\Omega^2 + \nu X\left[-\Omega\hat{h}_u + \hat{\sigma}_y + \hat{\sigma}_z\right]. \end{aligned} \tag{32}$$

These equations can then be written in the form (23) and (24).

Supplementary Note 2: further details of numerical simulation algorithms

2.1 Procedure for adding pedestrians on the bridge

Initially the bridge-pedestrian system is simulated with a crowd size of two pedestrians. Every T_{add} of simulation time, we insert another pedestrian with a uniform random phase initialised to the limit cycle solution of the model in the absence of bridge motion. Subsequently, we advance the simulation T_{add}

seconds, compute the maximum amplitude of the bridge and mean order parameter of the pedestrians over the current interval, and save the entire state.

2.2 Implementation of negative damping criterion

To compute σ_1: According to the formula (9), for each pedestrian, we need to compute

$$\bar{h}_u = \frac{1}{T}\int_0^T \frac{\partial H}{\partial u} dt,$$

where $u = \dot{x}$. We consider specifically the case of Models 1 and 3 in which there is a jump in the force H. Specifically, for $t \in (t_{s-1}, t_{s+1})$ we can write

$$H(y,t) = H_s(y) + \Theta(t - t_s)J(y; t_s, t_{s-1}),$$

where Θ is the Heaviside step function,

$$H_s(y) = \sqrt{\frac{g}{L}}\left(p(t_{s-1}) - y(t)\right), \quad J(y(t); y(t_s), \dot{y}(t_s)) = \sqrt{\frac{g}{L}}\left(p(t_s) - p(t_{s-1})\right)$$

and $p(t_s)$ is given by (13). The principle is easily generalised to any function $H(y)$ with jumps at $t = t_s$. That is H during step s is given by H_s and $J = H_{s+1} - H_s$. For the specific case of Models 1 and 2 we can write

$$\frac{\partial H}{\partial u} = \frac{\partial H}{\partial y}\frac{\partial y}{\partial u} + \frac{\partial H}{\partial t}\ddot{x} = -\sqrt{\frac{g}{L}}\frac{\partial y}{\partial u} \quad + \delta(t - t_s)J(t_s)\ddot{x}.$$

Hence

$$\int_{t_{s-1}^+}^{t_{s+1}^-} \frac{\partial H}{\partial u} dt = J(y(t_s)) - \int_{t_{s-1}^+}^{t_{s+1}^-} \sqrt{\frac{g}{L}}\frac{\partial y}{\partial u}(t)\, dt\,.$$

That is, we compute the integral as if the singularity were absent, provided we add an extra jump term every time $t = t_s$ for $s = 1, 2, 3, \ldots$

It remains to show how to compute

$$\eta(t) := \frac{\partial y}{\partial u}(t) \qquad \text{from } t_{s-1} \text{ to } t_s.$$

Note that

$$\frac{d^2\eta}{dt^2} = \frac{\partial}{\partial u}\left(\frac{\partial^2 y}{\partial t^2}\right)$$
$$= \frac{\partial}{\partial u}(-\ddot{x} - H(y)) = -\dot{u}\,\ddot{u} - \frac{\partial H}{\partial y}\eta.$$

Hence, $\eta(t)$ satisfies the variational differential equation

$$\ddot{\eta} = \sqrt{\frac{g}{L}}\eta - \ddot{u}\,\dot{u} \qquad \text{subject to} \quad \eta(t_{s-1}) = \dot{\eta}(t_{s-1}) = 0. \tag{33}$$

To compute σ_1 for the particular case of Model 3, we proceed similarly; in this case, note that the jump term

$$\delta(y)J(y,\dot{y},t) = \delta(y)\lim_{\varepsilon\to 0}[-\lambda^2\nu^2\dot{y}(y(t+\varepsilon) - p_c\mathrm{sgn}(y(t+\varepsilon)))^2 + \frac{g}{L}(y(t+\varepsilon) - p_c\mathrm{sgn}(y(t+\varepsilon)))$$
$$+\lambda^2\nu^2(y - p_c\mathrm{sgn}(y(t))^2 - \frac{g}{L}(y(t) - p_c\mathrm{sgn}(y(t)))]$$
$$= \delta(y)\left[4\lambda^2\nu^2 p_c y\mathrm{sgn}(\dot{y}) + 2\frac{g}{L}(p_c\mathrm{sgn}(\dot{y}))\right] = \delta(y)\left[2\frac{g}{L}(p_c\mathrm{sgn}(\dot{y}))\right]$$

is due to the presence of the discontinuity in $\mathrm{sgn}(y)$ at each step and takes a similar form to that of the jump term of Model 1 in the case of a fixed step width p_c.

Likewise, we may formulate a second-order variational equation, akin to (33), for the continuous part of $H_{\dot{x}}$ using the multivariate chain rule

$$\dot{\eta}(t) - \dot{u}\ddot{u} - \eta H_y - \dot{\eta} H_{\dot{y}}, \quad \text{where} \quad \eta(t) = \partial_u y(t).$$

Within the pedestrian step, $H_{\dot{y}}$ and H_y have closed form and may be used to compute σ_2 (see next section).

To compute σ_2. Here, according to (10), for each pedestrian we need to compute y_c y_s, $\overline{h}_y$ and $\overline{h}_v$. By definition, y_c and y_s would be the Fourier cosine and since coefficients, respectively, of the $O(\varepsilon)$ components of $y(t)$, assuming that the dominant bridge motion is $\cos(\Omega t)$. Thus, given bridge motion $x(t)$,

we have

$$y_c = \frac{1}{AT}\int_0^T x(t)y(t)dt, \qquad y_s = -\frac{1}{\Omega AT}\int_0^T \dot{x}(t)y(t)dt, \tag{34}$$

where

$$A^2 = \int_0^{2\pi/\Omega} x(t)^2 dt.$$

The integrals $\overline{h}_y$ and $\overline{h}_v$ can be computed similarly to $\overline{h}_u$. In particular, for Models 1 and 3 we get

$$\frac{\partial H}{\partial y} = \frac{dH}{dy} + \frac{\partial H}{\partial t}\ddot{y} = -\sqrt{\frac{g}{L}} + \delta(t-t_s)J(t_s)\dot{y}$$

and

$$\frac{\partial H}{\partial v} = \frac{\partial H}{\partial y}\frac{\partial y}{\partial v} + \frac{\partial H}{\partial t}\ddot{y} = 0 + \delta(t-t_s)J(t_s)\ddot{y}.$$

Hence,

$$\overline{h}(y) = -\sqrt{\frac{g}{L}} + \frac{1}{t_s - t_{s-1}}J(t_s)\dot{y}(t_s)$$

and

$$\overline{h}u = \frac{1}{t_s - t_{s-1}}J(t_s)\ddot{y}(t_s).$$

To compute σ_3. The formula (11) requires evaluation of the forward dynamics, specifically a function $z^{(i)}(t)$ which is the fluctuating component of the pedestrian's progress $Z^{(i)}(t)$

$$Z^{(i)}(t) = \chi^{(i)}t + z^{(i)}(t),$$

where $\chi^{(i)}$ is their average forward velocity. However, none of the models we consider have an equation for the forward dynamics $z(t)$.

Instead, Models 2 and 3 have an update rule that generates the time t_{s+1} as a function of t_s. Because everything is averaged over a cycle, we can assume that $z(t)$ is a continuous variable that at $t = t_s$ gives the perturbation to the position along the bridge span of the pedestrian's centre of mass from its position if it were walking at a constant velocity.

That is, we can assume that for $t \in [t_s, t_{s+1})$

$$\dot{z} = w_s, \qquad \text{where} \quad w_s = \frac{t_{s-1} - 2t_s + t_{s+1}}{t_{s+1} - t_s}$$

so that z becomes a piecewise-linear function of time

$$z(t) = z(t_s) + w_s(t - t_s), \qquad \text{for } t_s \le t < t_s + 1.$$

Then the functions z_c and z_s can be defined similarly to y_c and y_s above. Also

$$\frac{\partial H}{\partial z} = \frac{\partial H}{\partial t}\frac{\partial z}{\partial t} = \delta(t - t_s)J(t_s)w \quad \text{for} \quad t = t_s$$

and

$$\frac{\partial H}{\partial w} = \frac{\partial H}{\partial t}\frac{\partial w}{\partial t} = 0.$$

Hence, we need only consider the first term in (11), which necessitates computation of z_s. Analogously, with (34), we have

$$\overline{z_s} = -\frac{1}{\Omega A T}\int_{t_{s-1}}^{t_s} w\dot{x}(t)t\,dt.$$

2.3 Calculations of the scatter plots and analytical curves in Fig. 4.

To generate the scatter plots Fig. 4 (top row) and estimate average damping coefficient per pedestrian $\bar{\sigma}$, we simulate the pedestrian system (5) with imposed bridge motion, wherein the bridge is taken to be sinusoidal at its natural frequency, i.e.

$$x(t) = X_b \sin \Omega t,$$

where $X_b = 0.006$ m is the constant amplitude of the imposed bridge motion. Only a single pedestrian is placed on the bridge and the bridge's acceleration is fixed to the second derivative of the sinusoid: $\ddot{x} = -X_b \Omega^2 \sin \Omega t$, regardless of the pedestrian's foot force. This scenario accurately describes the case for a low mass ratio, such that the force from an individual pedestrian has negligible effect on the structure. We perform this simulation over a uniform 1200-point grid of the pedestrian and bridge natural frequencies $\overline{\omega}$ and Ω, where Ω ranges from 0.25 to 3 Hz and $\overline{\omega}$ ranges from 0.6 to 1.2 Hz. At each point of the grid we compute the mean frequency of the pedestrian and bridge movement. This numerically calculated frequency ratio $[\Omega/\overline{\omega}]$ may differ from the ratio of natural frequencies $\Omega/\overline{\omega}$ due to the adaptation of the pedestrian stride to the bridge motion. This yields the non-uniform distribution of the blue points across parameter range $[\Omega/\overline{\omega}]$ in Fig. 4 as the pedestrian frequency adaptation promotes specific frequency ratios of $[\Omega/\overline{\omega}]$.

For each frequency ratio $[\Omega/\overline{\omega}]$, we numerically compute the force $H^{(1)}$ by simulating equation (5) for $\ddot{y}_1$ over 50 foot steps for Model 2 and over 10,000 steps for Model 3. To calculate the component of $H^{(1)}$ in phase with the bridge velocity, we modify the formula (9) from Ref.[2] so that

$$\hat{H}_u = \frac{2}{t_s - t_{s-1}} \int_0^{t_s - t_{s-1}} H^{(1)} \cos \Omega t \, dt.$$

We then compute a histogram of $\hat{H}_u$, parametrised by a discrete set of K numerically computed phase offsets φ of the pedestrian step time. To calculate the damping coefficient of the pedestrian, we use the histogram to calculate the expected value $\overline{H}_u$ of $\hat{H}_u$ for each φ. We then apply the scaling in equation (11) from Ref.[2] to the resulting average. Thus, we obtain the following formula

$$\overline{\sigma} = -\frac{\overline{H}_u}{X_b \Omega} = -\frac{2}{K X_b \Omega (t_s - t_{s-1})} \sum_{k=1}^{K} \int_0^{t_s - t_{s-1}} H^{(1)} \cos \Omega t \, dt, \tag{35}$$

where the summation is calculated over the discrete bins of the histogram. This formula is used to generate the blue points in the top panels of Fig. 4 for different frequency ratios $[\Omega/\overline{\omega}]$.

The analytical curve for $\overline{\sigma}$ depicted by the green solid line in the top plot of Fig. 4 (a) is calculated via

$$\overline{\sigma} = \frac{mg}{L} A^2 \frac{\overline{\omega}}{\pi\Omega^2} \left(\Omega \sqrt{\frac{L}{g}} a_2 - a_1 \right), \tag{36}$$

where $A = \frac{1}{1+\frac{g}{L\Omega^2}}$, $a_1 = 1 - e^{\frac{\pi}{\overline{\omega}}\sqrt{\frac{g}{L}}} \cos \frac{\pi\Omega}{\overline{\omega}}$, and $a_2 = e^{\frac{\pi}{\overline{\omega}}\sqrt{\frac{g}{L}}} \sin \frac{\pi\Omega}{\overline{\omega}}$.

Formula (36), derived in Ref.[3], estimates the negative damping contribution of the pedestrian described by Model 1 via $\overline{\sigma}_1$. As a result, it does not account for the effect of step timing adaptation and, therefore, it yields estimates that differ from the numerical results for Model 2 that does allow for pedestrian step timing adaptation.

We also use formula (36) to estimate the critical crowd N_{crit} as a function of the frequency ratio Ω/ω (the green solid line in the bottom plot of Fig. 4 (a)). This is done by setting the total bridge damping $c_T = 0$ in (8). Therefore, we obtain the condition

$$2\varepsilon\zeta\Omega + N_{crit}\overline{\sigma}/M = 0$$

which yields the following estimate for the critical crowd size

$$N_{\mathrm{crit}} = -\frac{2\varepsilon\zeta\Omega M}{\overline{\sigma}}, \tag{37}$$

where $\overline{\sigma}$ is estimated via (36). The analytical expression (37) estimates the critical number of pedestrians N_{crit} described by Model 1 precisely. However, it becomes less accurate for pedestrians described by Model 2, and especially by Model 3 in which $\sigma_2^{(i)}$ plays a significant role (see Supplementary Fig. 1 for the comparison of the damping terms $\sigma_1^{(i)}$ and $\sigma_2^{(i)}$ in Models 2 and 3). For this reason, the curve (37) is plotted in the bottom plot of Fig. 4 (a) for Model 1 only.

Each blue point in the bottom plots of Fig. 4 indicates a critical number of pedestrians N_{crit} for a combination of Ω and $\overline{\omega}$ for each ratio $[\Omega/\overline{\omega}]$. This critical number N_{crit} is numerically determined as a fixed crowd size which makes the bridge amplitude grow and exceed 0.006 m within a time period of

$50\pi/\overline{\omega}$ s. These simulations were performed for the same bridge parameters as in Fig. 3 but for identical pedestrians with the same angular stride frequency ω, varied across the range of frequency ratio $[\Omega/\overline{\omega}]$, and the same default values of b_{min}, L, and m given in Table 3. As in the simulations of Fig. 3, the initial conditions for the pedestrian phases were chosen randomly from a uniform distribution. Zero bridge amplitude and velocity were chosen as the initial conditions for the bridge.

Supplementary Note 3: further simulation results

3.1 Faster addition of pedestrians to the bridge

To better understand the role of time interval T_{add} at which pedestrians are added sequentially to the bridge, we perform numerical simulations similar to those reported in Fig. 3 but with shorter time interval $T_{\text{add}} = 10$ s. In this case, the pedestrian-bridge system has a narrower time window for transient effects before the addition of the next pedestrian. As a result, one can expect that the crowd will have grown larger by the time the vibrations have increased in amplitude significantly. The simulations displayed in Supplementary Fig. 1 confirm this intuition and indicate the widening of the instability region (pink) preceding the onset of weak (Model 2) and strong synchronisation (Model 3).

To elucidate the contributions of the damping per pedestrian terms σ_1, σ_2, σ_3, we also include the additional plots (third row) in Supplementary Fig. 1. These plots indicate that while σ_1 is the only factor that matters for the onset of instability for non-adaptive Model 1, σ_2, that accounts for the adjustment of pedestrian lateral gait timing, contributes to the overall negative damping to a lesser (Model 2) and greater (Model 3) degree. In all cases we find that σ_3 makes little contribution.

3.2 Extreme worst-case, complete resonance

To provide further evidence that pedestrian synchronisation is not necessary for bridge instability, we consider the worst-case scenario in which the pedestrians have identical natural stride frequency ω (but

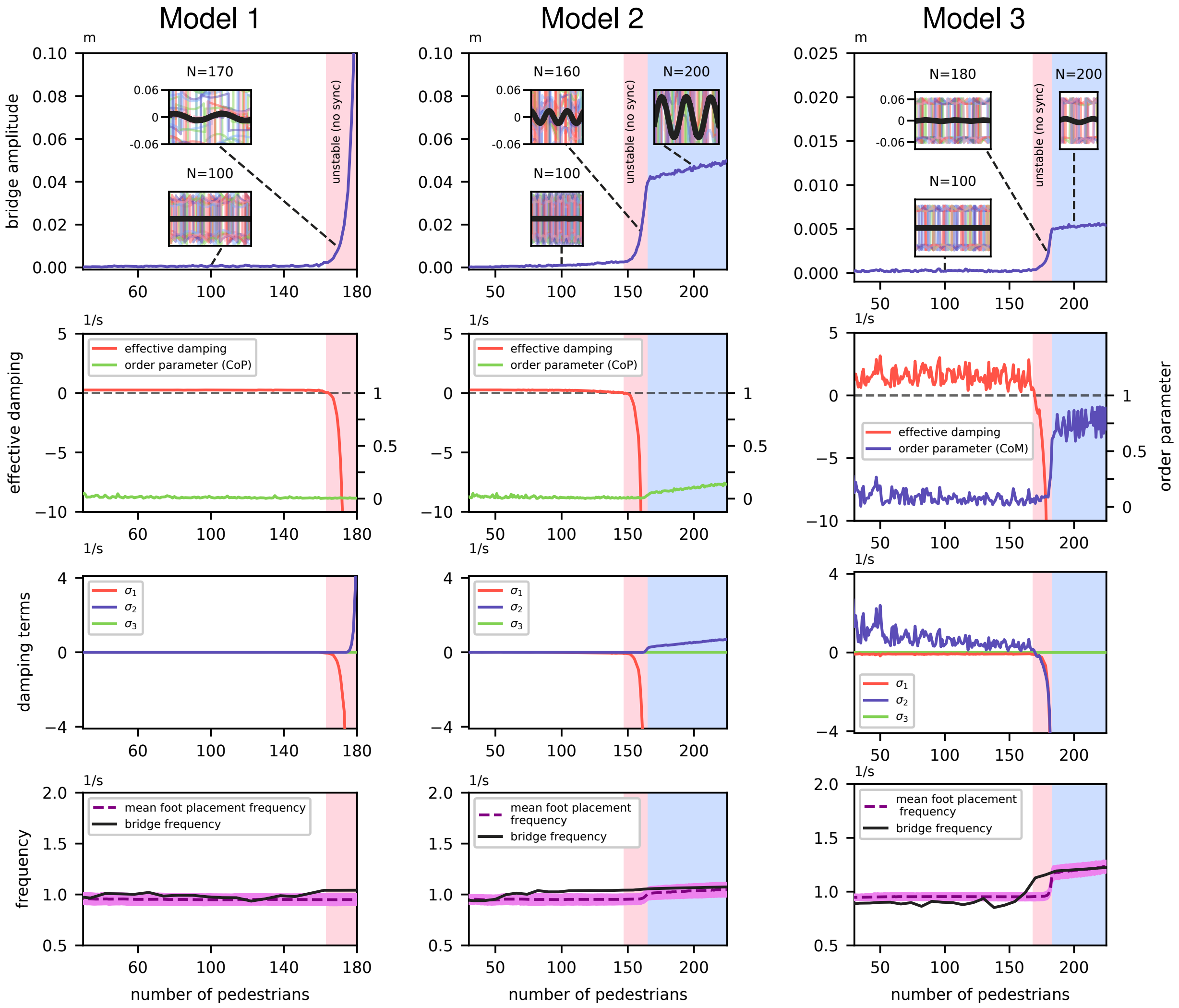

Supplementary Figure 1. Simulations as in Fig. 3 in which pedestrians are sequentially added at shorter intervals of $T_{\mathrm{add}} = 10$ s. Notice the widening of the pink region corresponding to the onset of bridge oscillations without pedestrian phase locking. The plots for the damping per pedestrian terms $\sigma_{1,2,3}$ specify the contribution of each term to the onset of bridge instability. Other parameters are as in Fig. 3.

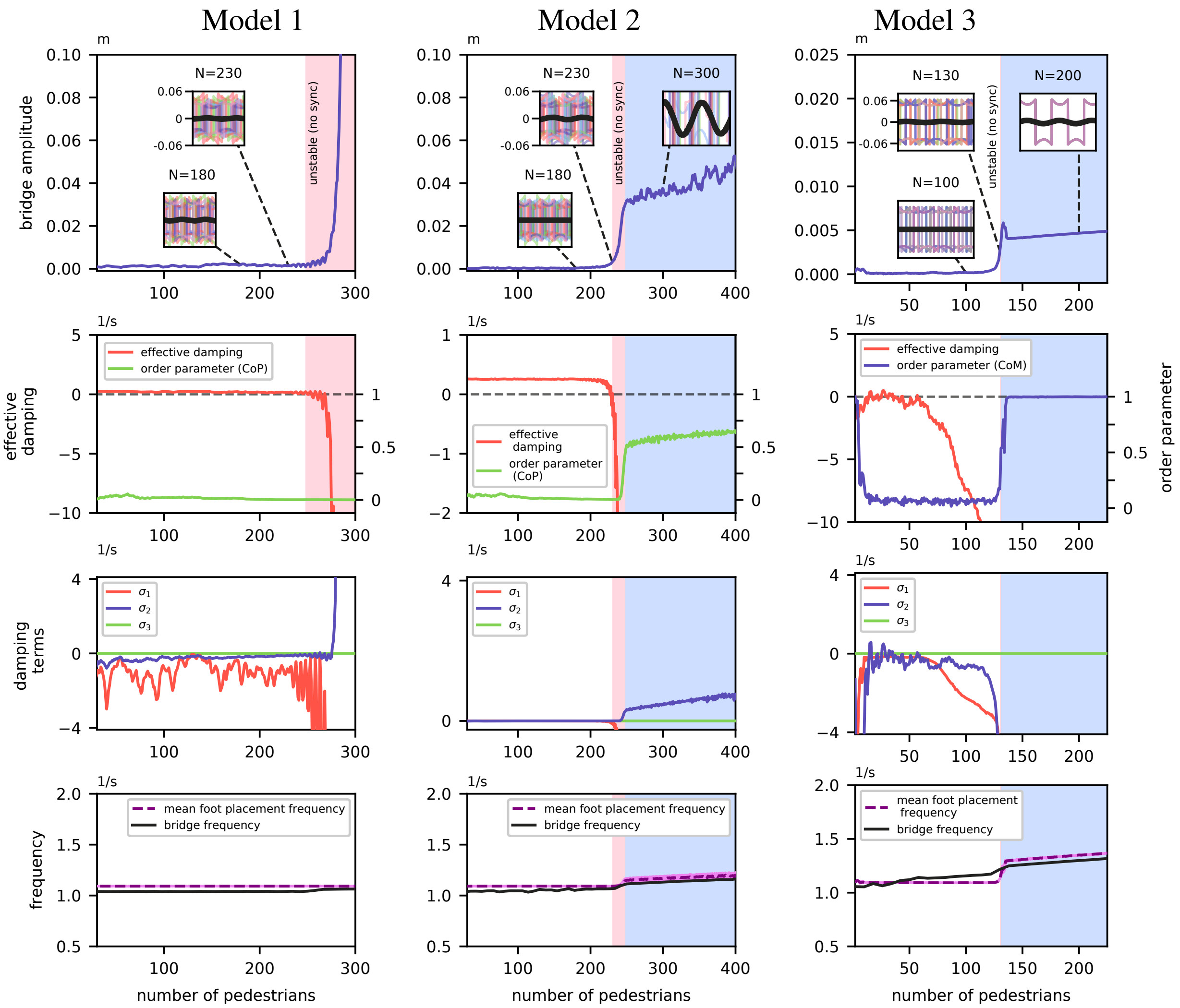

Supplementary Figure 2. Simulations as in Fig. 3 but for the worst case scenario of pedestrians with the same $\omega = 5.655$ rad/s ($S.D. = 0$) and the perfect resonance ratio $\Omega/\omega = 1$ corresponding to the yellow point in Fig. 4. Other parameters are as in Fig. 3 .

initially random phase) which also coincides with the bridge natural frequency Ω. It seems to be the ideal resonance scenario for the emergence of synchronisation among the pedestrians and with the bridge, and therefore, we could expect synchronisation to emerge at smaller crowd sizes and coincide with the onset of bridge instability. We also note that this case violates the central assumption (21) that underlies the above asymptotic derivation of the coefficients $\sigma_{1,2,3}$.

The results are shown in Supplementary Fig. 2. Despite violating the above assumption, observe that the negative damping criterion still predicts the onset of bridge instability for Models 1 and 2. In particular, note how significant synchronisation now occurs, after the onset of large vibrations, for Model 2 with an order parameter greater than 0.5. For Model 3, the negative damping criterion no longer provides accurate information, but note that the large increase in negative damping at around 50-100 pedestrians precedes the onset of significant instability, which in this case leads to complete synchrony (order parameter $r = 1$).

Supplementary References

1. A.H. Nayfeh. *Perturbation Methods*. Wiley, Chichester, UK, 2000.

2. M. Bocian, J.H.G. Macdonald, and J.F. Burn. Biomechanically inspired modelling of pedestrian-induced forces on laterally oscillating structures. *Journal of Sound and Vibration*, 331(16):3914–3929, 2012.

3. A. McRobie. Long-term solutions of Macdonald's model for pedestrian-induced lateral forces. *Journal of Sound and Vibration*, 332:2846–2855, 2013.
