# Supplementary material for: Emergence of the London Millennium Bridge instability without synchronisation
Source: Nat Commun. 2021 Dec 10;12:7223. doi: 10.1038/s41467-021-27568-y (PMC8664840; doi:10.1038/s41467-021-27568-y)
Supplement: Supplementary file 3 — Description of additional Supplementary File [file 41467_2021_27568_MOESM3_ESM.pdf]

Description of additional Supplementary Data Files

File Name: Supplementary Movie 1
Description: Displays a pedestrian walking according to Model 2 subject to an imposed bridge motion (see Methods for the details).
